# Theoretical and Experimental Study on Nonlinear Failure of an MEMS Accelerometer under Dual Frequency Acoustic Interference

**DOI:** 10.3390/s21030945

**Published:** 2021-01-31

**Authors:** Peng Guo, Jiayu Zhang, Lihui Feng, Jianmin Cui, Chaoyang Xing

**Affiliations:** 1 The Key Laboratory of Photonics Information Technology, Ministry of Industry and Information Technology, School of Optics and Photonics, Beijing Institute of Technology, Beijing 100081, China; guopeng0304@bit.edu.cn (P.G.); 3120180597@bit.edu.cn (J.Z.); cueijianmin@bit.edu.cn (J.C.); 2 Beijing Institute of Aerospace Control Device, Beijing 100094, China; mems13@163.com

**Keywords:** MEMS accelerometer, acoustic injection, nonlinear effects, short-time Fourier transform, resonance frequency

## Abstract

In order to quantitatively study the interfered output of the accelerometer under an acoustic injection attack, a mathematical model for fitting and predicting the accelerometer output was proposed. With ADXL103 as an example, an acoustic injection attack experiment with amplitude sweeping and frequency sweeping was performed. In the mathematical model, the R-squared coefficient was *R*^2^ = 0.9990 in the acoustic injection attack experiment with amplitude sweeping, and *R*^2^ = 0.9888 with frequency sweeping. Based on the mathematical model, the dual frequency acoustic injection attack mode was proposed. The difference frequency signal caused by the nonlinear effect was not filtered by the low-pass filter. At a 115 dB sound pressure level, the maximum acceleration bias of the output was 4.4 m/s^2^ and the maximum amplitude of fluctuation was 4.97 m/s^2^. Two kinds of methods of prevention against acoustic injection attack were proposed, including changing the damping ratio of the accelerometer and adding a preposition low-pass filter.

## 1. Introduction

Micro-Electro-Mechanical Systems (MEMS) are a high-tech frontier subject. They have been developed on the basis of integrating various micromachining technologies and applying the latest achievements of modern information technology. The MEMS inertial sensor as the main component of the MEMS system has very broad application prospects [1,2,3,4], including a MEMS gyroscope and MEMS accelerometer. Therefore, the reliability of MEMS inertial sensors is worthy of attention [5,6,7]. MEMS inertial sensors are supposed to maintain stable and accurate outputs in any harsh environment. However, if there is an acoustic signal close to or identical to the resonant frequency in the MEMS inertial sensor, due to the design structure of the spring-mass-damping system, it outputs the wrong data. The output of MEMS inertial sensors can even be controlled by modulating the frequency of sound waves [8,9,10,11].

This feature has aroused great interest in recent years. In 2014, Yan Michalevsky et al., of Stanford University, processed the built-in MEMS gyroscope signal of mobile phones via machine learning, restoring voice information [12]. In 2015, Yunmok Son et al., from the Korea Academy of Science and Technology, introduced the working status of Unmanned Aerial Vehicles (UAVs) equipped with MEMS gyroscopes under resonant acoustic interference [13]. In 2017, Trippel et al. from the University of Michigan, studied sound wave interference on the output of MEMS accelerometer, which was caused by the failure of amplifiers or low-pass filters [14]. In 2017, an Alibaba security scientific research team presented the experimental results for acoustic waves’ destructive interference with MEMS inertial devices, which caused the system to stop working, at the Black Hat Security Technology Conference [15]. Shadi Khazaaleh et al., (2019), of New York University, evaluated the vulnerability of MEMS gyroscopes to ultrasonic attacks on targets. The main cause of ultrasonic shock is the disharmony between the gyroscope’s sensitive axis and driving axis [16]. In 2019, the Hui Li scientific research team of Wuhan University verified the influence of acoustic interference on a MEMS accelerometer through ANSYS multiphysical field simulation [17].

According to Trippel et al., (2017) [14], the failure of the accelerometer under acoustic injection is mainly due to amplifier failure or low-pass filter failure. However, there is no quantitative mathematical model for the nonlinear output caused by amplifier failure. Therefore, we proposed a mathematical model that can fit the experimental data. This mathematical model can predict the nonlinear effect of acoustic signals on the accelerometer output. This mathematical model provides some references for acoustic wave controllable attacks on a MEMS accelerometer and antiacoustic wave interference on MEMS devices.

Based on this mathematical model of the amplifier, we proposed a dual frequency acoustic attack method. According to the nonlinear effect, by injecting two sound waves, the difference frequency signal cannot be filtered by a low-pass filter.

## 2. Acoustic Injection Experiment

### 2.1. Acoustic Injection Experiment Facility

**Figure 1:**
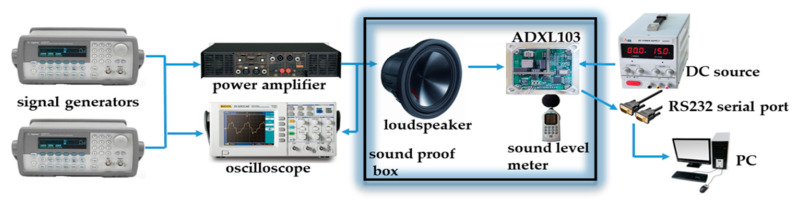
Acoustic injection; experimental facility.

In order to verify the influence of acoustic interference on the MEMS accelerometer, an experimental platform was built to conduct real measurements [18,19,20]. The experimental test platform of the MEMS accelerometer with acoustic interference consists of three parts: the first subsystem generates sound signals, the second subsystem monitors real-time signals (including observing input sound signals, sound signals output by power amplifier, and sound pressure level), and the third subsystem acquires the MEMS accelerometer’s output signals. The basic schematic diagram of the experimental test platform of the MEMS accelerometer with acoustic interference is shown in Figure 1.

In the experiment, two functional signal generators (Agilent 33250A) were used to generate electrical signals. They were passed to the input of the power amplifier (AE TECHRON 7224) and then fed the amplified electrical signal into the loudspeaker. The loudspeaker was SV220 and the sound frequency range was 1~12 kHz. An oscilloscope (RIGOL DS1102E) was used to observe the input waveform of the signal generator. The output waveform was amplified by the power amplifier. A sound level meter (HT-850A) was used to measure the sound pressure level (SPL) at a point in the experimental sound field. The MEMS accelerometer ADXL103 was fixed to its Printed Circuit Board (PCB) matters only by means of soldered connections, and the output signal of the MEMS accelerometer was directly read by the official software on the PC through the RS232 serial port. To isolate the external sound source interference, we placed the MEMS accelerometer and loudspeaker packages in a soundproof box. The established acoustic injection experiment facility met the acoustic frequency of 1~12 kHz, and the sound pressure level (SPL) reached 120 dB.

The typical product of the capacitive MEMS accelerometer selected in the experiment was ADXL103 from the ADI company’s uniaxial MEMS accelerometer. Its full range acceleration measurement range was ± 1.7 g (g: gravity acceleration). Typical background noise (110 μg/√Hz, 5 mm, 5 mm, 2 mm, 8 pin ceramic LCC package) was provided. According to the datasheet of ADXL103, the sensor resonant frequency is about 5500 Hz [21].

The experiment was carried out in a constant temperature laboratory with a temperature of 25 ± 3 °C, and the zero g bias temperature coefficient of ADXL103 is less than 0.3 mg/°C. The maximum output bias due to thermal drift is 1.8 mg, far less than the acceleration bias caused by acoustic injection. The thermal drift of the accelerometer is negligible. We calibrated the output of the accelerometer before each experiment, and the duration of every experiment was less than 600 s. The long term drift of the accelerometer is negligible too.

### 2.2. Acoustic Injection Experiment with Frequency Sweeping

**Figure 2:**
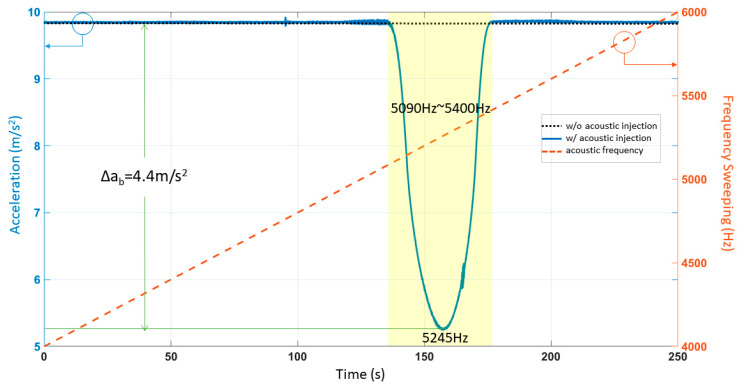
The output acceleration of ADXL103 under the acoustic injection attack of frequency sweeping.

We performed the acoustic injection attack of sinusoidal frequency sweeping on ADXL103 to determine the resonant frequency. The signal generator was conducted by sweeping a sinusoidal signal from 4000 Hz to 6000 Hz, which took 250 s. The oscilloscope observed the peak-to-peak voltage of the signal generator (3.0 V), and the peak-to-peak voltage of the power amplifier (20.8 V). The distance between the sound source and the ADXL103 was about 10 cm, and the sound pressure level (SPL), measured by the sound pressure meter, was around 115 dB. We set the sensitive axis to vertical. The time-domain responses of the experimental results are shown in Figure 2. In order to visually determine the resonant frequency range of the ADXL103, the range of the sweep-frequency is shown by the orange line in Figure 2. According to the time-domain image, we determined that the resonant frequency range of the ADXL103 was 5090 Hz–5400 Hz. When the interference was most severe, the sound frequency was 5245 Hz. The absolute value of the difference between the measured acceleration without the acoustic injection and with the single frequency acoustic injection is defined as *∆a_b_*. The maximum value was 4.4 m/s^2^. The previous acoustic injection attack accelerometer experiment resulted in an offset of 1.27 m/s^2^ [17]. We achieved 3.46 times the previous result. 

### 2.3. Acoustic Injection Experiment with Amplitude Sweeping

**Figure 3:**
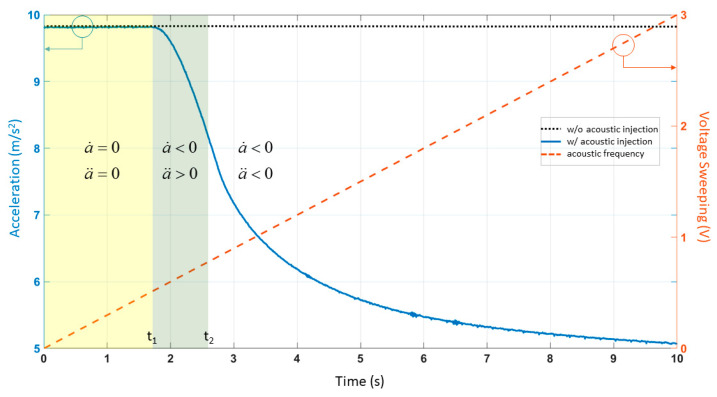
The output acceleration of ADXL103 under the acoustic injection attack with amplitude sweeping at frequency of 5245 Hz.

The output of ADXL103 is shown in Figure 2. The big change in the output above 5245 Hz indicated the resonant frequency. We kept the frequency constant during the acoustic injection experiment with amplitude sweeping. We set the peak-to-peak voltage of input to 0 V–3 V. The period of amplitude sweeping signal was 10 s; the max sound pressure level was about 115 dB, and the distance between the sound source and the accelerometer was about 10 cm. The experimental results are shown in Figure 3. 

After analyzing the experimental results of the acoustic injection attack with amplitude sweeping, we noticed that it did not fall linearly. We found that there were two inflection points at 1.81 s (*t*_1_) and 2.59 s (*t*_2_) when we combined the experimental results to find the second derivative, $\ddot{a}$. The acceleration output was not disturbed before *t*_1_. The acceleration between *t*_1_ and *t*_2_ dropped rapidly, i.e., with first derivative $\dot{a}$ *<* 0 and second derivative $\ddot{a}$ < 0. After *t*_2_, the acceleration dropped slowly, i.e., with first derivative $\dot{a}$ *<* 0 and second derivative $\ddot{a}$ *>* 0.

## 3. A Mathematical Model of the Accelerometer with Acoustic Injection Attacks

### 3.1. A Mathematical Model for Bilateral Asymmetric Clips

According to the known research of MEMS accelerometers with acoustic injection attacks, the main output error is typically caused by amplifier or low-pass filter failure [14]. The experimental results show that the output error of ADXL103 is caused by the amplifier with bilateral asymmetric clips. 

On the one hand, the process of acoustic injection attacks on the accelerometer is very complicated [22,23]. The acoustic wave also vibrates on some additional mechanical elements, such as the support upon which the accelerometer PCB rests. On the other hand, the signal output from the accelerometer is also subjected to complex electrical processing, including capacitance converters, amplifiers, low-pass filters, and demodulators [21]. Therefore, we propose a mathematical model to fit the process. It not only avoids complex physical models and circuit analysis but it also accurately describes the output of the accelerometer under acoustic injection attacks.

**Figure 4:**
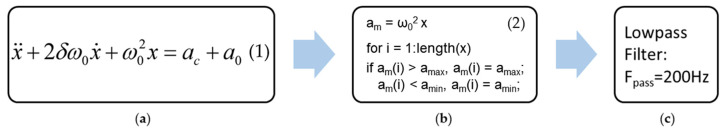
The three steps of the mathematical model: (**a**) An equation used to calculate the output value. (**b**) The output with bilateral asymmetric clips. (**c**) A low-pass filter.

The mathematical model includes three steps, as shown in Figure 4:

Step 1: We first needed the Figure 4a to simulate the output of the accelerometer (Figure 4a). *x* is a function of *t*. *a_c_* represents practical effects of the acoustic injection on the accelerometer. *a*_0_ represents the measured acceleration without the acoustic injection. To compare that with the experimental results, set *a*_0_ = 9.8 m/s^2^. δ is the damping ratio. ω_0_ is the natural frequency.

Step 2: Bilateral asymmetric clips of *a_m_*. 

Step 3: Signals with frequency less than 200 Hz can pass. Since the low-pass frequency of ADXL103 used in the experiment was 200 Hz, we had better fitting results.

The setting of simulation parameters was shown in Table 1. Analysis of the acoustic injection experiment result of frequency sweeping determines *δ* and ω_0_^2^ by the resonant frequency $f=\omega_{0}\sqrt{1-\delta^{2}}$. In Figure 2, the experimental results of the sweeping frequency acoustic injection attack showed that the resonant frequency was 5245 Hz. Analysis of the acoustic injection experiment result with amplitude sweeping determined *a_max_* and *a_min_* via *t*_1_ and *t*_2_. 

### 3.2. The Mathematical Model Fit with Acoustic Injection Experiment with Amplitude Sweeping

**Figure 5:**
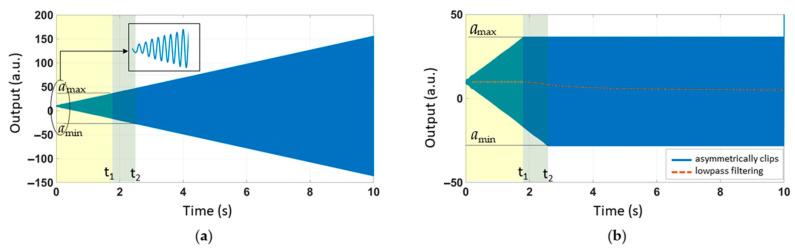
(**a**) The time-domain response of linear amplitude sweeping. (**b**) Bilateral asymmetric clipping and low-pass filtering.

In the acoustic attack experiment with amplitude sweeping, *a_c_ = B · t ·* sin*(2πft)* is the relevant equation. *f* is the resonant frequency of the accelerometer (5245 Hz). *B* represents a magnification factor: *B* = 2.04 m/s^3^. The numerical solution of differential equation was calculated using the ODE45 function solver in MATLAB. Figure 5a shows the time-domain response. *t*_1_ is the corresponding coordinate, *a_max_. t*_2_ is the corresponding coordinate, *a_min_.* We set the bilateral asymmetric clipping range to *a_max_*~*a_min_*. The results are shown in blue in Figure 5b. The output of the low-pass filter was underlined in orange in Figure 5b. The low-pass filter was allowed to pass at a frequency below 200 Hz. This step was calculated using the FDATOOL in MATLAB.

**Figure 6:**
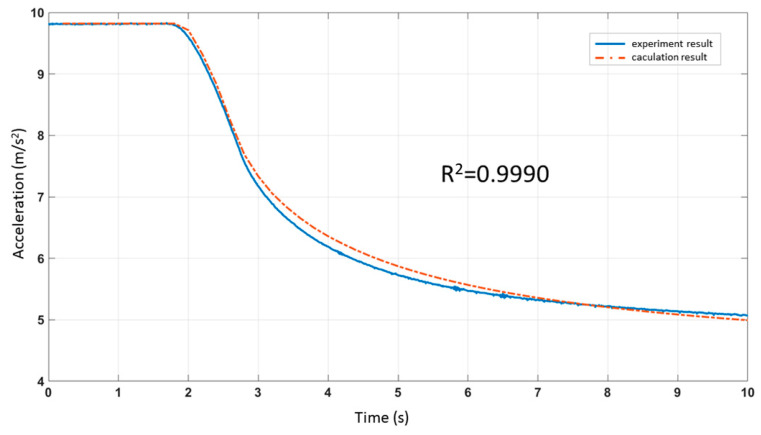
Comparison of the experimental data and mathematical model results under the sweeping amplitude acoustic attack.

After time normalization, the output of the filter (Figure 5b) was compared with the experimental results (Figure 3), as shown in Figure 6. The R-squared coefficient *R^2^* was used to represent the fitting degree of simulation and experiment. We called the function corrcoef in MATLAB, *R^2^ =* 0.9990.

### 3.3. The Mathematical Model Fit with Acoustic Injection Experiment with Frequency Sweeping

**Figure 7:**
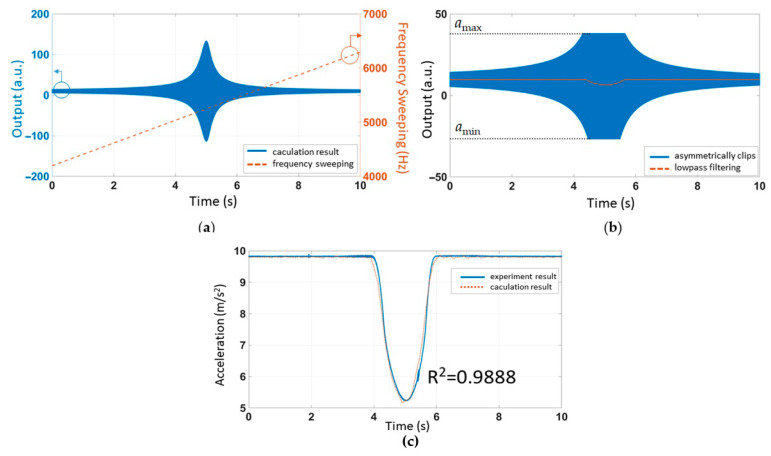
(**a**) The time-domain response of linear frequency sweeping. (**b**) Bilateral asymmetric clipping and low-pass filtering. (**c**) Comparison of experimental data and mathematical model results under the sweeping frequency acoustic attack.

In the acoustic attack experiment with amplitude sweeping, we set *a_c_ = B ·* sin[*2πf*(*0.8t + 0.02t*^2^)]. The sweeping frequency ranged from 0.8*f* to 1.2*f*. It took 10 s to complete the process. Signal clipping occurred when the output exceeded the range of the amplifier and the first-order low-pass filter was realized via data averaging. After setting the appropriate simulation parameters, the simulation results were plotted. The time-domain response of linear frequency sweeping was plotted in Figure 7a. We set the amplifier’s bilateral asymmetric cutoff range to *a_min_*~*a_max_*. In Figure 7b, the blue line represents the amplifier’s failure and the orange line shows the detailed result after the LPF. The simulation results in Figure 7b were compared with the experimental results in Figure 2 and then plotted in Figure 7c. The R-squared coefficient *R^2^* was used to represent the fitting degree of the simulation and experiment. After MATLAB calculation, we found that *R^2^ =* 0.9888.

## 4. Simulation of the Dual Frequency Acoustic Injection Attack

Based on this mathematical model, we proposed a dual frequency acoustic attack method. Due to the nonlinear effect, dual frequency acoustic signals produce sum and difference frequency signals. The difference frequency signals cannot be filtered by a low-pass filter.

**Figure 8:**
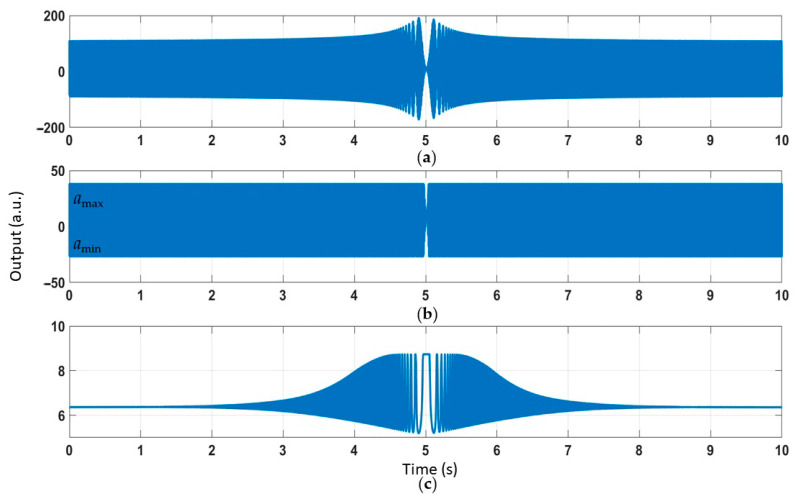
(**a**) The time-domain response of dual frequency acoustic injection. (**b**) Bilateral asymmetric clipping. (**c**) Low-pass filtering.

We set *a_c_ = B ·* sin[*2πf*(*0.8t + 0.02t*^2^)] *+ B ·* sin(*2πft*). In the simulation, one of the sound waves was single frequency, which was the same as the resonant frequency, *f.* The other sound wave was the sweeping frequency from 0.8*f* to 1.2*f*. It took 10 s to complete the process. The output time-domain response is shown in Figure 8. Figure 8a–c corresponds to the three steps in the mathematical model in Figure 4.

**Figure 9:**
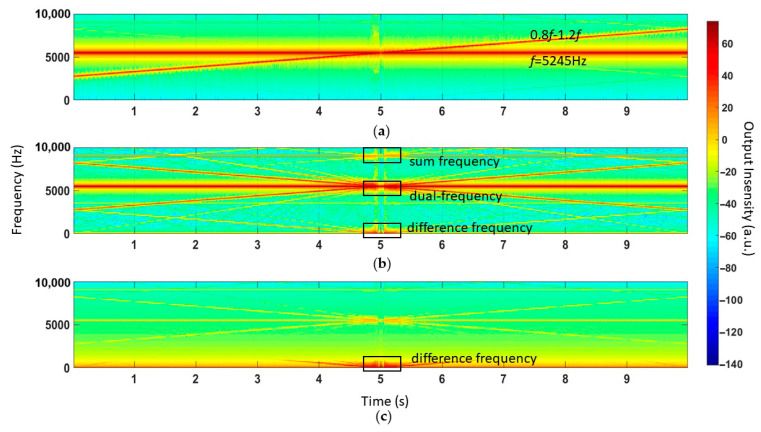
(**a**) Injecting dual frequency acoustic attack, an acoustic signal with fixed frequency and an acoustic signal with sweeping frequency. (**b**) The nonlinear effect leads to the generation of sum frequency signals and difference frequency signals. (**c**) Low-pass filtering only preserves the difference frequency signal.

The short-time Fourier transform (STFT) was used to determine the frequency of the output; the time-frequency response is shown in Figure 9. The original signal contained two types of frequency information, as shown in Figure 9a: an acoustic signal with fixed frequency *f* and an acoustic signal with sweeping frequency 0.8*f*~1.2*f*. Because of the bilateral asymmetric clipping, the nonlinear effect led to the generation of sum frequency signals and difference frequency signals (Figure 9b). Finally, in Figure 9c, low-pass filtering only preserved the difference frequency signals.

## 5. Experiment of Dual Frequency Acoustic Injection Attack

### 5.1. Acoustic Injection Attack Experiment with Two Superimposed Single Frequencies

**Figure 10:**
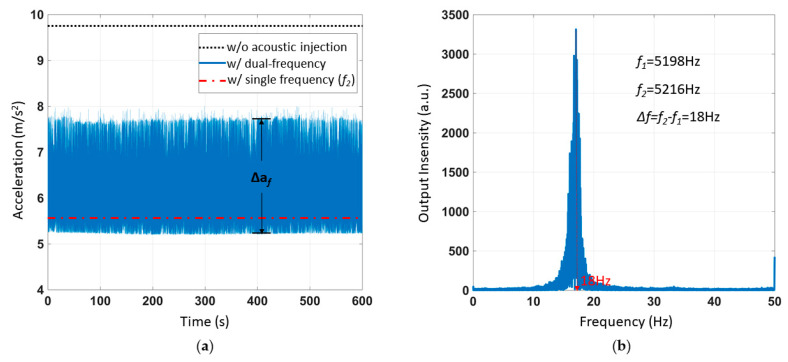
Two single frequency (*f*_1_ = 5198 Hz, *f*_2_ = 5216 Hz) acoustic injection attacks on ADXL103. (**a**) The acceleration of the ADXL103 output. (**b**) The result of the fast Fourier transformation (FFT).

During the experiment, the ADXL103 uniaxial accelerometer measuring axis was vertical. The measured acceleration was 9.8 m/s^2^ without acoustic interference. Firstly, the MEMS accelerometer ADXL103 was interfered with via two single frequency sound waves at different frequencies, for verifying the feasibility of the experiment’s dual frequency interference. One of the sound waves had a frequency of 5198 Hz; the other was 5216 Hz. In the experiment, the SPL was 115 dB. The official software sampling frequency of ADXL103 was 100 Hz. The test time was 600 s. Figure 10a shows the acceleration of ADXL103 output, which was different from the DC output under the single frequency sound wave interference. The output under the interference of two single frequency sound waves was AC. In the same experimental setup, the output results with only the single frequency sound waves interference and no sound wave, respectively, were represented by red and black lines in Figure 10a. The fast Fourier transformation (FFT) was applied to the output result, and the result is shown in Figure 10b. The frequency was 18 Hz, which was the difference frequency between the two sound waves. The acceleration of the amplitude fluctuation caused by the AC signal was recorded as *Δa_f_ =* 2.6 m/s^2^. There was no generation for the acceleration of the amplitude fluctuation in previous research [17].

### 5.2. Acoustic Injection Attack Experiment of Single Frequency and Sweeping Frequency Superimposing

**Figure 11:**
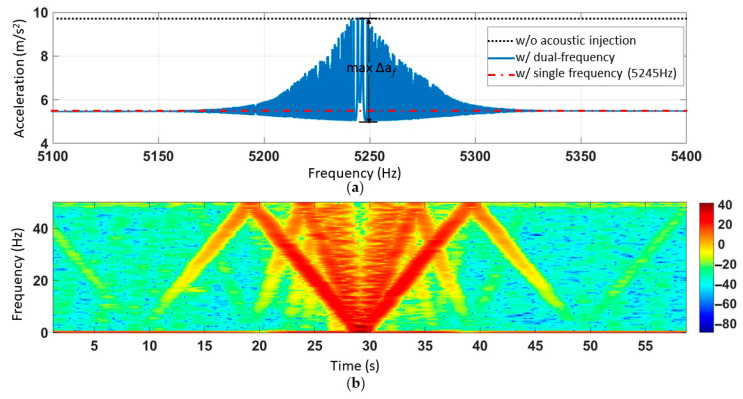
The acceleration of ADXL103 interfered by acoustic injection of superimposed resonant frequency and sweeping frequency. (**a**) Acceleration output of acoustic injection during the experiment. (**b**) Time-frequency response.

Then, a signal generator was setup to output 5.245 kHz single frequency sound waves. Another signal generator was set to the output sinusoidal sweeping frequency sound waves with a range of 5.1 kHz~5.4 kHz and a sweeping time of 60 s. The amplification factor of the power amplifier was kept constant, and the SPL (115 dB) was measured by the sound level meter. The experimental results are shown in Figure 11a. In the same experimental setup, the output results when there was only single frequency interference with no sound wave were represented by red and black lines, the difference of which was *∆a_b_* = 4.4 m/s^2^. The maximum fluctuation amplitude (*∆a_f_*) was 4.8 m/s^2^. The short-time Fourier transform of the output acceleration is shown in Figure 11b. Because of the official ADXL103 software sampling frequency was 100 Hz, according to the Nyquist sampling theory, the sampling frequency of STFT would be 50 Hz. When the frequency exceeded 50 Hz, the line folded down. As shown in Figure 11b, the frequency of the output result corresponded to the difference frequency between 5.245 kHz and sweeping frequency. Due to the nonlinear effect of the system, the output results contain not only the difference frequency signal, but also the frequency-doubled and frequency-tripled signals of the difference frequency signal. The darkest red line represents the difference frequency signal, with the frequency-doubled and frequency-tripled signals fading in turn.

### 5.3. Acoustic Injection Attack Experiment of Two Sweeping-Frequency Superimposing

**Figure 12:**
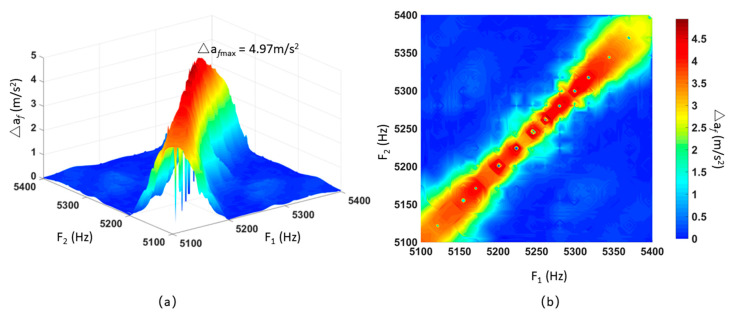
The amplitude of fluctuation for the MEMS accelerometer ADXL103 output under dual sweeping frequency acoustic injections. (**a**) Three-dimensional image. (**b**) Top view.

There are two signal generators in the experimental facility. One signal generator outputs different fixed frequency signals between 5.1 kHz and 5.4 kHz. For each fixed frequency signal, the other signal generator is sweeping from 5.1 kHz to 5.4 kHz. The output signal measured by the oscilloscope prevailed. The SPL (115 dB) was kept unchanged during all experiments. The ADXL103 output accelerometer was recorded and the experimental results were interpolated. Figure 12a shows that when dual frequency sound waves interfered with ADXL103 acceleration, the output produced the magnitude of fluctuation amplitude Δ*a_f_.* The X-axis and Y-axis represented the acoustic interference frequency output by difference signal generators, while the Z-axis represented the amplitude of the fluctuation caused by acceleration. Figure 12b is a top view of Figure 12a to better observe the results. The maximum amplitude of fluctuation was 4.97 m/s^2^.

The experimental results in Figure 10a correspond to the points that *F_1_* = 5198 Hz, *F_2_* = 5216 Hz, Δ*a_f_* = 2.6 m/s^2^ in Figure 12b. The experimental results in Figure 11a correspond to the line that *F_1_* = 5245 Hz in Figure 12b.

## 6. Discussion

In this section, two improved methods were proposed to protect the MEMS accelerometer from acoustic interference. The principle is as follows, and the experiment will be carried out in the future research.

### 6.1. MEMS Accelerometers with Different Damping Ratios

**Figure 13:**
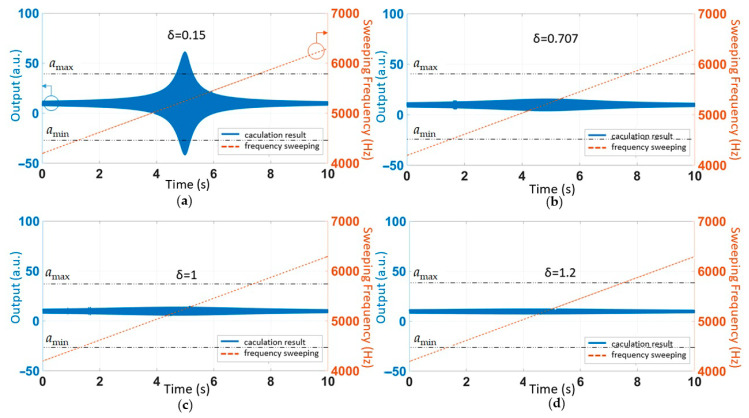
The accelerometer’s output in the case of different damping ratios. (**a**) *δ* = 0.15, underdamped system. (**b**) *δ* = 0.707, underdamped system. (**c**) *δ* = 1, critically damped system. (**d**) *δ* = 1.2, overdamped system.

The damping ratio parameter *δ* of the system determines the transient response characteristics of the MEMS accelerometer system. The smaller the damping ratio *δ* is, the more strongly it is affected by resonant frequency interference. We changed the value of the damping ratio in the proposed mathematical model (Figure 4), analyzing the relationship between the accelerometer response and the sound frequency under different conditions. The remaining parameters were unchanged. The results are shown in Figure 13.

According to the simulation results in Figure 13, it can be concluded that when the MEMS accelerometer system was in an underdamped state, the output had obvious responses to the swept sound wave near the resonant frequency. When the MEMS accelerometer system was critically damped or overdamped, the output of the MEMS accelerometer was almost unaffected by the swept sound wave near the resonant frequency. The amplitude of the signal did not reach the bilateral asymmetric clip. The effect of the acoustic injection was completely eliminated by a low-pass filter. Therefore, in the case of acoustic interference, increasing the damping of the system can effectively avoid the bilateral asymmetric cut-off of the amplifier. However, too much damping reduces the accuracy of the accelerometer [19]. Therefore, this method may be suitable for accelerometers that operate in complex acoustic fields and have low accuracy requirements.

### 6.2. Preposition Low-Pass Filter

**Figure 14:**
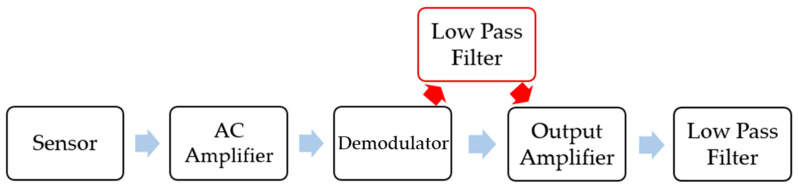
A preposition low-pass filter.

The Analog Devices Inc. released the circuit design [24,25,26] as shown in Figure 14. The output voltage of the sensing part is once amplified and demodulated, then twice amplified and low-pass filtered.

To filter out the extra acceleration caused by the sound waves, we tried adding another low-pass filter between the demodulator and the output amplifier. The measured acceleration signal without the acoustic injection was a DC signal. Acoustic acceleration had a high frequency signal and the low-pass filter filtered the acoustic acceleration. Although this method avoids the acoustic acceleration that causes failure of the amplifier, it increases the complexity of the circuit and increases cost.

## 7. Conclusions

Taking accelerometer ADXL103 as an example, we injected an acoustic attack into it, and found that only an acoustic frequency matching the mechanical resonant frequency would affect the output of the accelerometer. There are two main reasons for the failure of the accelerometer by acoustic interference: amplifier failure and low pass filter failure [14]. By observing the experimental results, we judged that the acoustic interference of the ADXL103 output is mainly caused by the failure of the amplifier and we proposed a mathematical model that can fit the experimental data. In the fitting process, the calculated results of the mathematical model were experimentally fitted. The R-squared coefficient reached *R^2^* = 0.9990 in the acoustic injection attack experiment with amplitude sweeping. The R-squared coefficient reached *R^2^* = 0.9888 in the acoustic injection attack experiment with frequency sweeping. Then, we carried out the experiment of a dual frequency acoustic injection attack on ADXL103. Compared to a single frequency acoustic attack, the maximum offset of the output error acceleration (∆*a_b_)* was 4.4 m/s^2^ and the maximum fluctuation amplitude (∆*a_f_*) was 4.97 m/s^2^. In the experimental results of the existing single frequency attack accelerometer, the maximum offset of the single frequency acoustic interference was 1.27 m/s^2^ without amplitude fluctuation [17]. Firstly, the interference amplitude of the dual frequency attack greatly improved. Secondly, as a new means of interfering with MEMS devices, it presented new challenges for protection.

## Figures and Tables

**Table 1:** Parameters.

Parameter Name (unit)	Symbol	Parameter Value
Damping ratio	*δ*	0.07
Natural frequency (rad/s)	ω_0_	33037.9
Maximum value that can be output (a.u.)	*a_max_*	38.26
Minimum value that can be output (a.u.)	*a_min_*	−26.72

## Data Availability

Data sharing not applicable.
